# Recent Updates on the Pathogenesis of Inflammatory Myopathies

**DOI:** 10.1007/s11926-024-01164-7

**Published:** 2024-09-24

**Authors:** Jon Musai, Andrew L. Mammen, Iago Pinal-Fernandez

**Affiliations:** 1 Muscle Disease Section, National Institute of Arthritis and Musculoskeletal and Skin Diseases, National Institutes of Health, 50 South Drive, Room 1141, Building 50, MSC 8024, Bethesda, MD 20892 USA; 2 Department of Neurology, Johns Hopkins University School of Medicine, Baltimore, MD USA

**Keywords:** Myositis, Pathogenesis, Autoantibodies, Dermatomyositis, Immune-mediated Necrotizing Myositis, Inclusion body Myositis, Antisynthetase Syndrome

## Abstract

**Purpose of Review:**

This review aims to provide a comprehensive and updated overview of autoimmune myopathies, with a special focus on the latest advancements in understanding the role of autoantibodies. We will begin by examining the risk factors and triggers associated with myositis. Next, we will delve into recent research on how autoantibodies contribute to disease pathogenesis. Finally, we will explore the latest innovations in treatment strategies and their implications for our understanding of myositis pathogenesis.

**Recent Findings:**

Recent research has revealed that myositis-specific autoantibodies can infiltrate muscle cells and disrupt the function of their target autoantigens, playing a crucial role in disease pathogenesis. Significant advances in treatment include CD19 CAR-T cell therapy, JAK-STAT inhibitors, and novel strategies targeting the type 1 interferon pathway in dermatomyositis. Additionally, the ineffectiveness of complement inhibitors in treating immune-mediated necrotizing myositis has challenged established views on disease mechanisms.

**Summary:**

Autoimmune myopathies are a collection of disorders significantly influenced by specific autoantibodies that drive disease pathogenesis. This review highlights the critical role of autoantibody research in deepening our understanding of these conditions and discusses recent therapeutic advancements targeting key pathogenic pathways.

## Introduction

Autoimmune myopathies are a heterogeneous family of autoimmune diseases that target multiple organs, including muscle, skin, lungs, and/or joints [1]. These diseases have been grouped into clinical syndromes such as dermatomyositis (DM), antisynthetase syndrome (AS), immune-mediated necrotizing myopathies (IMNM), and inclusion body myositis (IBM) [1].

**Fig. 1:**
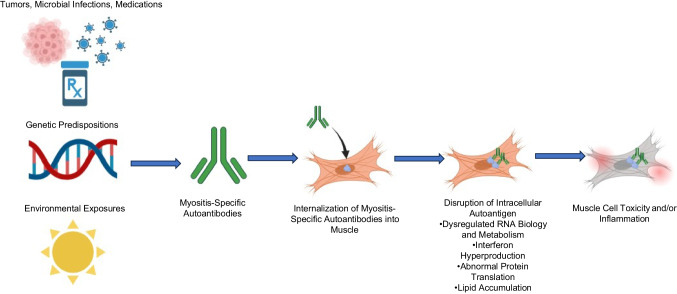
Proposed pathogenesis of idiopathic inflammatory myopathies. The development of myositis is influenced by a complex interplay of pre-existing diseases, microbial infections, medications, genetic predispositions, and environmental exposures. These factors may contribute to the production of myositis-specific autoantibodies, which have been found to accumulate within the muscle tissue of patients. Recent research has linked the pathogenesis of myositis to the intracellular actions of these autoantibodies, disrupting the function of their specific autoantigens. This disruption may contribute to muscle cell toxicity and inflammation.

Our understanding of the pathogenesis of myositis has evolved significantly over the years due to epidemiologic advances and molecular research. A critical advancement has been the discovery of multiple autoantibodies that define groups of myositis patients with unique clinical features, prognosis, and response to treatment [1, 2]. It is well established that most myositis patients have a known autoantibody targeting a specific intracellular autoantigen. For instance, dermatomyositis autoantigens include Mi-2 [3], TIF1ꝩ [4], NXP2 [5], MDA5 [6], or SAE [7]; antisynthetase syndrome patients have autoantibodies recognizing one of the aminoacyl-tRNA synthetases including Jo1 [8], PL7 [9], and PL12 [10, 11]; immune-mediated necrotizing myopathy patients have autoantibodies recognizing either SRP [12] or HMGCR [13]; and autoantibodies recognizing EXOSC 9/10 (anti-PM/Scl) are linked to overlap myositis [14]. Recent efforts have focused on identifying the pathogenesis of different myositis autoantibodies by analyzing their specific clinical, histopathological, and molecular differences. Additionally, studies on various types of iatrogenic inflammatory myopathy, including those induced by treatment with checkpoint inhibitors —chemotherapies that remove the brakes on the immune system— [15, 16] or bone marrow transplantation resulting in graft-versus-host disease [17], have further enhanced our understanding of the mechanisms underlying myositis. (Figure 1)

In this review, we will discuss recent findings related to the pathogenesis of myositis. We will begin by discussing the risk factors and disease triggers of myositis, followed by an analysis of the autoantibodies and immune processes involved in modulating inflammation and causing damage. Lastly, we will review the latest advancements in treatments and how they have contributed to a deeper understanding of myositis pathogenesis.

## Predisposing Factors and Triggers

The rarity and heterogeneity of myositis have made it difficult to investigate the predisposing factors, yet several epidemiological factors have been associated with a higher risk of developing an inflammatory myopathy. Dermatomyositis, antisynthetase syndrome, and overlap myositis are more prevalent in females, with the highest incidence occurring in childhood and middle age [18, 19]. In contrast, inclusion body myositis is more common in males, typically manifesting in late adulthood [18]. Immune-mediated necrotizing myopathy is more prevalent in females, with a median onset in middle to late adulthood, although it can also affect children [18].

Regarding ethnic differences, epidemiological studies have documented that anti-MDA5 dermatomyositis is more commonly observed in East Asian populations [20, 21]. Also, anti-HMGCR immune-mediated necrotizing myositis has been recently found to be ~ 150-fold more prevalent in American Indians receiving statins compared to the general population [22]. 

Concerning environmental risk factors, regions with higher ultraviolet radiation intensity, such as those closer to the equator, exhibit a higher relative frequency of dermatomyositis compared to other types of myositis [23]. Additionally, exposure to dust and other environmental pollutants has been linked to an increased risk of developing various forms of myositis [24–26]. In patients with anti-MDA5 dermatomyositis, interstitial lung disease primarily occurs from October to March [20, 21], suggesting that seasonal respiratory viruses may influence disease pathogenesis. Other viruses, such as HIV [27], HCV [28], and HTLV-1 [29], have also been associated with different forms of myopathy, though their roles in disease pathogenesis remain poorly understood. 

Unlike viral infections, the association between cancer and dermatomyositis is well-established [18]. Dermatomyositis patients with anti-TIF1ꝩ autoantibodies have a particularly high risk of malignancy [4, 30, 31], while those with anti-NXP2 autoantibodies have a significant but lower risk [5]. Whole-exome sequencing of patients with anti-TIF1γ cancer-associated myositis revealed somatic mutations and loss of heterozygosity in TIF1 genes [30, 31]. These data suggest that mutations in TIF1 genes may trigger an immune response against the mutated proteins in the tumor, leading to the production of anti-TIF1 autoantibodies. However, tumors can evade destruction through loss of heterozygosity to delete the mutated DNA sequence, thereby redirecting the immune response, including anti-TIF1γ autoantibodies, to target tissues with heightened TIF1γ expression, such as muscle and skin [30, 32]. Recently, it has been discovered that a proportion of anti-TIF1ꝩ-positive dermatomyositis patients have additional autoantibodies, including those recognizing Sp4 and CCAR1, which are negatively associated with the risk of malignancy in these patients [33, 34]. One hypothesis to explain this phenomenon is that a robust immune response against mutated proteins in the tumor, resulting in the production of multiple autoantibodies (such as anti-Sp4 and anti-CCAR1), might prevent tumor cells from evading immune surveillance and could potentially lead to their complete eradication. However, further research is necessary to fully understand this phenomenon.

In addition to cancer, certain drugs increase the risk of autoimmunity [35]. For instance, statins have been conclusively linked to the risk of developing anti-HMGCR immune-mediated necrotizing myositis [36]. Although the mechanism remains poorly understood, it is known that the risk is higher for certain types of statins (e.g., atorvastatin [37]) and that this association is more pronounced in Western countries compared to Eastern countries [38], where dietary sources of statins (such as oyster mushrooms, Pu-erh tea, or red yeast rice) may be more common than the pharmacologic use of statins. It has been hypothesized that statins may modify the structure of HMGCR, leading to the generation of novel epitopes and the subsequent production of antibodies against the modified protein. Through epitope spreading, these antibodies might ultimately come to recognize native HMGCR. However, this model of how statins could break tolerance to HMGCR has yet to be confirmed.

Another class of drugs, immune checkpoint inhibitors (ICIs), which have revolutionized cancer treatment, can also trigger myositis [39, 40]. The exact mechanism underlying ICI-induced myositis remains to be elucidated. Furthermore, until recently, it was not known whether ICI-myositis was a single entity or a mix of conditions modified by checkpoint inhibitor-induced immune system activation. It was hypothesized that adverse immune-mediated events following cancer treatment are driven by pre-existing autoantibodies against tissue-specific autoantigens [41], the presence of expanded effector memory T lymphocytes [42], and/or the reactivation of chronic viral infections [43]. In thymoma patients undergoing ICI treatment, pre-existing autoantibodies against muscle acetylcholine receptors and low B cell frequencies prior to ICI treatment were linked to the development of myositis [44]. Of note, a minority develop a DM-like skin rash [15, 16, 45].

Bulk and single nuclei RNA sequencing have identified three transcriptomically distinct subsets of ICI-myositis: ICI-DM, ICI-MY01, and ICI-MY02. Muscle biopsies from these subsets overexpress IL6 pathway genes [16]. In contrast, only the ICI-DM subset has marked overexpression of type I IFN-inducible genes, as is seen in DM. In contrast, the ICI-DM and ICI-MY01 subsets overexpress type II IFN-inducible genes.

Histological analysis of muscle biopsy specimens revealed that each type of ICI-myositis had unique morphological features. While ICI-DM patients have perifascicular pathology, as seen in DM, ICI-MYO1 patients have highly inflammatory muscle biopsies and ICI-MYO2 patients have necrotizing muscle biopsies.

Furthermore, the different ICI-myositis subsets had different clinical features. For example, all cases of co-existing myocarditis were present among those with ICI-MYO1. In contrast, all of the patients with DM-like rashes were in the ICI-DM subset. Importantly, in some of these, anti-TIF1ꝩ autoantibodies were detectable before ICI treatment initiation, confirming prior findings that pre-existing autoantibodies likely contribute to ICI-myositis [16].

Taken together, these findings support checkpoint inhibitor-induced myositis as a heterogeneous set of conditions where immune hyperactivation, triggered by checkpoint inhibitors, is directed towards areas targeted by different types of autoantibodies, such as the muscle in ICI-myositis. Furthermore, they suggest that, beyond general immunosuppressants, therapies targeting the interferon pathway (e.g., JAK/STAT inhibitors) or the IL6 pathway—potentially enhancing checkpoint inhibitor efficacy [46]—, along with strategies to reduce circulating autoantibodies could be effective in managing this condition.

Recent reports of patients with another form of iatrogenic inflammatory myopathy—myopathy occurring in the context of graft-versus-host disease after bone marrow transplantation [17] —have revealed striking histological similarities to immune checkpoint inhibitor myositis [15, 16, 45]. This suggests the intriguing possibility of shared pathogenic mechanisms between immune checkpoint inhibitor myositis and GVHD.

Complementary and alternative medicine, including herbal supplements for skin conditions, has become increasingly popular [35, 47]. Nevertheless, herbs such as spirulina, aphanizomenon flosaquae, chlorella, echinacea, and alfalfa have been reported to harm patients with autoimmune skin diseases like dermatomyositis [35, 47]. A case series described three patients who experienced an acute onset or flare-up of dermatomyositis after taking a popular herb-based weight loss product [48]. In vitro testing revealed that this product caused a dose-dependent increase in the release of proinflammatory cytokines in peripheral blood mononuclear cells [48]. With the growing use of complementary and alternative medicine, future research should continue exploring how herbal supplements stimulate the immune system in patients with DM and other autoimmune diseases.

Finally, compelling evidence suggests a genetic predisposition in inflammatory myopathy patients [49, 50], primarily linked to HLA antigens. For instance, HLA-DRB1*11:01 is found in over two-thirds of adult anti-HMGCR patients, contrasting with less than 20% prevalence in the general population [51]. Similarly, specific HLA antigens have been identified in association with other types of autoantibodies [52, 53]. Type 2 HLA molecules have been proposed as crucial factors influencing the autoantibody repertoire, potentially by promoting the survival of specific autoantibody-producing cells [54]. If validated, the robust HLA associations observed in various autoantibody-defined subgroups of myositis could be intimately linked to the risk of developing certain types of autoantibodies.

## Role of Myositis Autoantibodies in Disease Pathogenesis

A breakthrough in understanding myositis pathogenesis occurred with the discovery of autoantibodies targeting cytoplasmic or nuclear antigens in patients. Despite each autoantibody being associated with a specific clinical phenotype, their role in myositis pathogenesis has been relatively unexplored due to the prevailing assumption that autoantibodies cannot penetrate living cells.

Transcriptomic studies lead to the identification of distinct transcriptomic signatures associated with specific autoantibody-defined forms of myositis [55–57].

Of those autoantibodies associated with specific transcriptomic profiles, muscle biopsies from patients with anti-Mi2 autoantibodies, which target subunits of the transcriptional repressor NuRD, exhibit a distinct gene expression profile characterized by the overexpression of a set of more than 100 genes enriched for those known to be suppressed by the Mi2/NuRD complex [57–60]. Research into the underlying cause of this specific transcriptomic pattern led to the discovery that these autoantibodies are deposited in the nuclei of muscle cells from dermatomyositis patients, where the Mi2 autoantigen resides [57, 58]. These findings suggest that anti-Mi2 autoantibodies exert a pathological effect by disrupting the function of the Mi2/NuRD complex within the nuclei of muscle tissue. Supporting this hypothesis, recent studies in which purified immunoglobulins from anti-Mi2 patients were internalized into cultured muscle cells induced the same anti-Mi2-specific gene overexpression observed in muscle biopsies of anti-Mi2 DM patients [60].

Anti-PM/Scl autoantibodies specifically target key components of the RNA exosome complex, EXOSC9 and EXOCS10 [14], which play a role in degrading various forms of RNA, including long non-coding RNAs and divergent transcripts [61, 62]. Applying techniques similar to those used to identify the pathologic role of anti-Mi2 autoantibodies, a recent study revealed that muscle tissue from patients with anti-PM/Scl autoantibodies has an aberrant accumulation of long non-coding RNAs and divergent transcripts [60].

Furthermore, muscle biopsies from these patients show antibodies deposited in the nucleolus [60], consistent with the subcellular localization of EXOSC9 and EXOSC10 [61, 62]. Finally, experiments involving the internalization of purified immunoglobulins from anti-PM/Scl patients into cultured muscle cells replicated the same pattern of gene overexpression observed in the muscle biopsies of these patients. Taken together, these findings indicate that anti-PM/Scl autoantibodies enter muscle cells and disrupt the function of the nuclear RNA exosome complex, leading to the accumulation of aberrant transcripts in muscle tissue.

There is also evidence of autoantibody internalization and the disruption of autoantigen function in muscle biopsies from patients with other myositis autoantibodies [60], . For instance, anti-MDA5 autoantibodies specifically target MDA5, a cytoplasmic sensor of viral RNA that initiates the activation of type I IFN pathways [63]. Muscle biopsies from anti-MDA5-positive patients reveal autoantibody deposition in the cytoplasm. Both these biopsies and cultured muscle cells internalized with purified antibodies from anti-MDA5 patients show significant overexpression of type I IFN-inducible genes, suggesting the possibility of direct activation of the MDA5 protein by these autoantibodies [55, 60]. In this regard, it is of note that a recent study demonstrated that anti-MDA5 autoantibodies target epitopes within the three enzymatically active helicase domains of MDA5 [64].

In addition to muscle tissue, heightened activation of type I IFN signaling and increased metabolic activities are observed in the lungs [65], skin [66], and peripheral B and T lymphocytes of anti-MDA5 patients [65]. While the effects of these autoantibodies on these tissues have not been extensively explored, similar mechanisms of autoantibody internalization and toxicity could potentially play a role in these extramuscular disease manifestations.

Antisynthetase autoantibodies target aminoacyl-tRNA synthetases, which load the appropriate amino acid onto the appropriate tRNA. These charged tRNAs then play an essential role in protein synthesis [67]. Among these, anti-Jo1, which targets histidyl-tRNA synthetase, is the most prevalent [8]. When antibodies from anti-Jo1 patients are internalized by cultured muscle cells, they induce a gene expression pattern similar to that observed in muscle biopsies from anti-Jo1 patients [60]. This includes the overexpression of genes such as EGR4 and CXCL8. Furthermore, in vitro inhibition of histidyl-tRNA synthetase using histidinol results in the increased expression of the same anti-Jo1-specific genes, including EGR4 and CXCL8. This provides evidence that anti-Jo1 autoantibodies disrupt histidyl-tRNA synthetase function within muscle tissue, a finding originally demonstrated in vitro [68].

HMG-CoA reductase (HMGCR) controls the rate-limiting step in cholesterol biosynthesis by converting HMG-CoA to mevalonate [63, 69]. Patients with anti-HMGCR autoantibodies have a form of myositis characterized by myofiber necrosis [11]. Unlike biopsies from myositis patients with most other myositis-specific autoantibodies, anti-HMGCR-positive muscle biopsies do not show a distinctive transcriptomic profile. However, the accumulation of lipids in myofibers is a distinguishing feature of these patients, similar to the lipid accumulation observed in patients treated with statins, which inhibit HMGCR [64]. Since anti-HMGCR autoantibodies specifically target the intracellular catalytic domain of HMGCR [13], we hypothesize that anti-HMGCR autoantibodies hinder the enzymatic ability of HMGCR. This inhibition may lead to the accumulation of acetyl-CoA upstream of HMGCR and subsequent accumulation of lipids, which could potentially exert toxic effects on myofibers. Recently, mutations in HMGCR which disrupt its function, have also been shown to cause a limb-girdle muscle dystrophy [70, 71].

## Type 1 Interferon in Dermatomyositis

Initial transcriptomic analyses of affected tissues from myositis patients revealed intense overactivation of the type 1 interferon pathway in dermatomyositis [72, 73], a finding that has been validated and expanded upon by multiple researchers [74, 75]. Follow-up studies confirmed similar levels of type 1 interferon activation among different types of autoantibodies in dermatomyositis. Additionally, significant activation of the type 2 interferon pathway was discovered in antisynthetase syndrome, inclusion body myositis, and dermatomyositis [56, 76].

Studies identifying autoantibody internalization as a relevant disease mechanism in myositis also pinpointed IFNβ1 as the main driver of type 1 interferon pathway activation in muscle tissue [60].

As mentioned earlier, recent experiments suggest that anti-MDA5 autoantibodies can activate the interferon pathway directly by entering muscle fibers and binding to their autoantigen [60]. For other dermatomyositis-associated autoantibodies, the mechanism of type 1 interferon activation is less clear. For example, although not proven, in anti-Mi2 dermatomyositis, IFN-β1 could be one of the genes derepressed by autoantibody-mediated disruption of the Mi2/NuRD complex. Recent research has identified NXP2 [77] and TIF1γ [77, 78] as potent inhibitors of the IFNβ1 gene expression. Preliminary evidence suggests these autoantibodies may trap their antigens in the cytoplasm, preventing them from inhibiting IFN-β1 expression [60], . Finally, the accumulation of undegraded RNA in patients with anti-PM/Scl autoantibodies may trigger the activation of the interferon pathway through the stimulation of antiviral intracellular mechanisms, such as RIG-I and MDA5.

As discussed later, recent therapeutic advances have confirmed and underscored the importance of the type 1 interferon pathway in dermatomyositis, translating this knowledge into actionable therapeutic options.

### Treatment Advances Related to Myositis Pathogenesis

Recent treatment trials have helped us to better understand myositis pathogenesis, either by seeming to confirm or contradict the importance of previously proposed disease mechanisms.

A series of studies had previously suggested that autoantibodies in immune-mediated necrotizing myositis damaged muscle cells by binding to undefined molecules on the surface of muscle cells and activating the complement cascade [79–81]. However, this theory has recently been challenged by a robust negative clinical trial in immune-mediated necrotizing myositis using the complement inhibitor Zilucoplan. In this study, the drug successfully blocked the activation of the complement cascade but had no effect on any measures of disease activity (i.e., creatine kinase) or the clinical outcomes of the patients [82].

As previously mentioned, the type 1 interferon pathway has emerged as a critical pathogenic mechanism in various forms of myositis. Over recent years, several drugs have been developed to target this pathway specifically: Dazukibart inhibits IFNB1 directly [83], while Anifrolumab targets its receptor [84]. Additionally, there are drugs that act more broadly on different points of the JAK-STAT pathway [85–88]. Promising results have been observed with various JAK-STAT inhibitors including ruxolitinib [85, 86], tofacitinib [87], and most recently baricitinib in juvenile dermatomyositis [88]. Recently released preliminary results from clinical trials of Dazukibart have shown significant efficacy [83]. Case reports and ongoing studies with Anifrolumab also show promise in this patient population [84]. However, due to the numerous agents targeting similar pathways, determining the most effective and least toxic option will be challenging, especially given the lack of direct comparative studies.

Currently available and widely used strategies to directly reduce the concentration of circulating autoantibodies include saturating the neonatal Fc receptor to decrease the half-life of endogenous autoantibodies by using intravenous immunoglobulin (IVIG). Alternatively, C20 + plasma cell precursors can be targeted by using anti-CD20 drugs such as rituximab. Recently, a large clinical trial using IVIG [89] confirmed the efficacy of this treatment approach for patients with dermatomyositis [90]. The largest clinical trial using rituximab did not reach the primary endpoint [91], but, in our clinical experience, we use rituximab in combination with IVIG to successfully treat the most refractory cases of inflammatory myositis. This approach, in our experience, is remarkably effective, with the added advantage that the IVIG decreases the potential immunosuppression due to decreased levels of immunoglobulin related to rituximab. Alternative options to saturate the Fc receptor are being tested, including drugs such as efgartigimod, which is currently used successfully in myasthenia gravis, another autoantibody-mediated autoimmune disease [92].

Regarding strategies to deplete or reset plasma cell precursors, in addition to anti-CD20, anti-CD19 drugs are now available and may also prove to be useful in myositis [93]. Moreover, CD19 CAR-T cell therapy is being explored to reset the antibody repertoire and achieve long-term, drug-free remission [94]. In a case series of three anti-Jo1 antisynthetase syndrome patients treated with CD19 CAR T cell therapy, all three patients experienced major clinical responses and complete resolution of symptoms [94]. These patients exhibited normalization of serum muscle enzymes and muscular function, along with cessation of extramuscular activity [94]. In a different case report, a patient with antisynthetase syndrome and refractory myositis and interstitial lung disease showed improvement in muscle and pulmonary function after CD19 CAR T cell therapy [95] and a patient with juvenile dermatomyositis reached a dramatic response after this treatment modality [96]. In addition to CD19-targeting CAR T cell therapy, CAR T cells targeting B cell maturation antigen (BCMA) have been utilized in myositis treatment [97]. Unlike CD19, BCMA is more selectively expressed within the B cell lineage, specifically targeting memory B cells, plasmablasts, and plasma cells [97]. BCMA-targeting CAR T cells were administered to a patient with refractory anti-SRP-positive IMNM. Following treatment, this patient showed a favorable safety profile and persistent clinical improvements [97]. In these patient cases, B cell reconstitution occurred with a reduction in disease-specific immunoglobulins, and there were no instances of high-grade cytokine release or neurotoxicity [94, 95, 97]. The resolution of symptoms in myositis patients following CAR T cell therapy confirms the translational research suggesting that disease-specific autoantibodies and autoreactive B cells may be key drivers of myositis pathogenesis [60].

The requirements for toxic premedication and the potential adverse events related to cytokine release syndrome, along with the increased long-term risk for hematologic malignancies due to blood product preparation, may limit the applicability of these therapies. However, engineering advances in the generation and administration of these treatments may enhance their usability and broaden their application.

Inclusion body myositis (IBM) remains the least well-understood form of inflammatory myositis. Anti-NT5c1a is the only autoantibody predominantly described in this group of patients [98, 99]. However, it is not specific for IBM and no definite link to a specific pathogenic mechanism has been discovered yet. Recent studies identified frequent terminally differentiated KLRG1-positive cells in this condition, often meeting the criteria for large granular lymphocytic leukemia [100]. Another proposed IBM-specific feature is the dysfunction of TDP43 and the appearance of cryptic exons [101], but this knowledge requires further validation and translation into clinical practice.

Clinical trials are underway to determine if targeting KLRG1 + cells [102] or using aggressive immunosuppressive strategies, such as sirolimus [103], can lead to sustained benefits in these patients. Currently, IBM is not only the least well-understood form of myositis but also the only one without effective treatment options.

## Conclusion

The pathogenesis of myositis is intricate, influenced by a complex interplay of environmental and genetic factors that collectively shape disease susceptibility. Recent advancements have increasingly associated the disease with the intracellular actions of pathogenic autoantibodies, disrupting the function of their specific autoantigens. Many critical questions remain unanswered, including the mechanisms of autoantibody internalization and the reasons for varying tissue susceptibility to autoantibody-mediated damage. It is also unclear whether similar mechanisms impact tissues and organs beyond muscle, and if these mechanisms are relevant to other autoimmune systemic diseases such as systemic sclerosis, vasculitis, or lupus. The precise roles of myositis-associated autoantibodies, such as anti-Ro52, require further elucidation, alongside lesser-studied antibodies like anti-SAE1. Moreover, exploring techniques to measure the biological activity of intracellular autoantibodies holds promise for gaining deeper insights into disease progression and the development of specific complications. Fortunately, as our understanding of myositis pathogenesis evolves, so too do therapeutic strategies targeting these pathogenic autoantibodies. Future research focused on refining these therapies for improved safety and efficacy will be essential not only for advancing treatment outcomes but also for validating translational hypotheses and enriching our comprehension of the disease.

## Data Availability

No datasets were generated or analysed during the current study.
